# Automatic diagnosis and measurement of intracranial aneurysms using deep learning in MRA raw images

**DOI:** 10.3389/fneur.2025.1544571

**Published:** 2025-04-24

**Authors:** Qingning Yang, Fengxi Chen, Li Li, Rong Zeng, Jiaqing Li, Jingxu Xu, Chencui Huang, Junbang Feng, Chuanming Li

**Affiliations:** ^1^Chongqing Key Laboratory of Emergency Medicine, Chongqing, China; Medical Imaging Department, Chongqing Emergency Medical Center, Chongqing University Central Hospital, School of Medicine, Chongqing University, Chongqing, China; ^2^Department of Radiology, 7T Magnetic Resonance Translational Medicine Research Center, Southwest Hospital, Army Medical University (Third Military Medical University), Chongqing, China; ^3^Pathology Department, Chongqing Emergency Medical Center, Chongqing University Central Hospital, School of Medicine, Chongqing University, Chongqing, China; ^4^Department of Radiology, The Second Affiliated Hospital of Chongqing Medical University, Chongqing, China; ^5^Department of Information, Chongqing Emergency Medical Center, Chongqing University Central Hospital, School of Medicine, Chongqing University, Chongqing, China; ^6^Department of Research Collaboration, R&D Center, Beijing Deepwise and League of PHD Technology Co., Ltd., Beijing, China

**Keywords:** magnetic resonance angiography, intracranial aneurysm, deep learning, diagnosis, measurement

## Abstract

**Background:**

The traditional procedure of intracranial aneurysm (IA) diagnosis and evaluation in MRA is manually operated, which is time-consuming and labor-intensive. In this study, a deep learning model was established to diagnose and measure IA automatically based on the original MR images.

**Methods:**

A total of 1,014 IAs (from 852 patients) from hospital 1 were included and randomly divided into training, testing, and internal validation sets in a 7:2:1 ratio. Additionally, 315 patients (179 cases with IA and 136 cases without IA) from hospital 2 were used for independent validation. A deep learning model of MR 3DUnet was established for IA diagnosis and size measurement. The true positive (TP), false positive (FP), false negative (FN), recall, sensitivity, and specificity indices were used to evaluate the diagnosis performance of MR 3DUnet. The two-sample *t*-test was used to compare the size measurement results of MR 3DUnet and two radiologists. A *p*-value of < 0.05 was considered statistically significant.

**Results:**

The fully automatic model processed the original MRA data in 13.6 s and provided real-time results, including IA diagnosis and size measurement. For the IA diagnosis, in the training, testing, and internal validation sets, the recall rates were 0.80, 0.75, and 0.79, and the sensitivities were 0.82, 0.75, and 0.75, respectively. In the independent validation set, the recall rate, sensitivity, specificity, and AUC were 0.71, 0.74, 0.77, and 0.75, respectively. Subgroup analysis showed a recall rate of 0.74 for IA diagnosis based on DSA. For IA size measurement, no significant difference was found between our MR 3DUnet and the manual measurements of DSA or MRA.

**Conclusion:**

In this study, a one-click, fully automatic deep learning model was developed for automatic IA diagnosis and size measurement based on 2D original images. It has the potential to significantly improve doctors’ work efficiency and reduce patients’ examination time, making it highly valuable in clinical practice.

## Introduction

Intracranial aneurysm (IA) is an abnormal saccular protrusion on the intracranial arterial wall. Rupture of IA is the third most common cerebrovascular accident disease, following cerebral thrombosis and hypertensive intracerebral hemorrhage (1, 2), with an annual incidence rate of approximately 1% (3). IA rupture is the primary cause of non-traumatic subarachnoid hemorrhage (SAH), accounting for 85% of all SAH cases, which have high mortality (approximately 50%) (4) and disability rate (approximately 10–20%) (5). If IA can be detected and diagnosed in a timely manner, preventive treatment can be taken to reduce the risk of rupture and avoid serious consequences. Therefore, early detection and diagnosis of IA are very important in clinical practice (6).

At present, the gold standard for diagnosing IA is digital subtraction angiography (DSA), while computed tomography angiography (CTA) is the most commonly used screening method in clinical practice. However, both are invasive examinations requiring contrast media and radiation exposure. Magnetic resonance angiography (MRA) is an alternative method to CTA and DSA that does not require invasion, contrast agents, or radiation exposure (7, 8). The traditional procedure of IA diagnosis and evaluation in MRA includes three steps: 3D reconstruction, IA identification, and size measurement. The entire process is manually operated, which is time-consuming and labor-intensive. Long processing times greatly affect the rapid diagnosis of IA, and the increased workload of radiologists may lead to missed diagnoses and serious consequences. Therefore, developing a fast and automated technology for the automatic diagnosis and size measurement of IA has important clinical significance.

In recent years, deep learning technology has developed rapidly and demonstrated significant value in the diagnosis and prognosis evaluation of many diseases. Previous studies have successfully applied it for the automatic reconstruction of head blood vessels and the automatic diagnosis of IA on CTA and DSA images (9–14). There were also studies using deep learning for IA recognition based on 3D reconstruction MRA images (15–20). However, until now, there was no artificial intelligence model to automatically perform IA diagnosis and size measurement on original 2D MR images. In this study, we collected samples from multiple centers and developed a deep learning model to automatically diagnose intracranial IA and measure its size based on original 2D MR images. We believe it would significantly help doctors improve their work efficiency and reduce patients’ examination time, which was of great significance for the early diagnosis and prognosis improvement of IA.

## Materials and methods

### Research participants

The study was approved by the Ethics Committee of our hospital (No. 2022(23), dated 26 April 2022). As this is a retrospective study, informed consent was not required.

**Figure 1:**
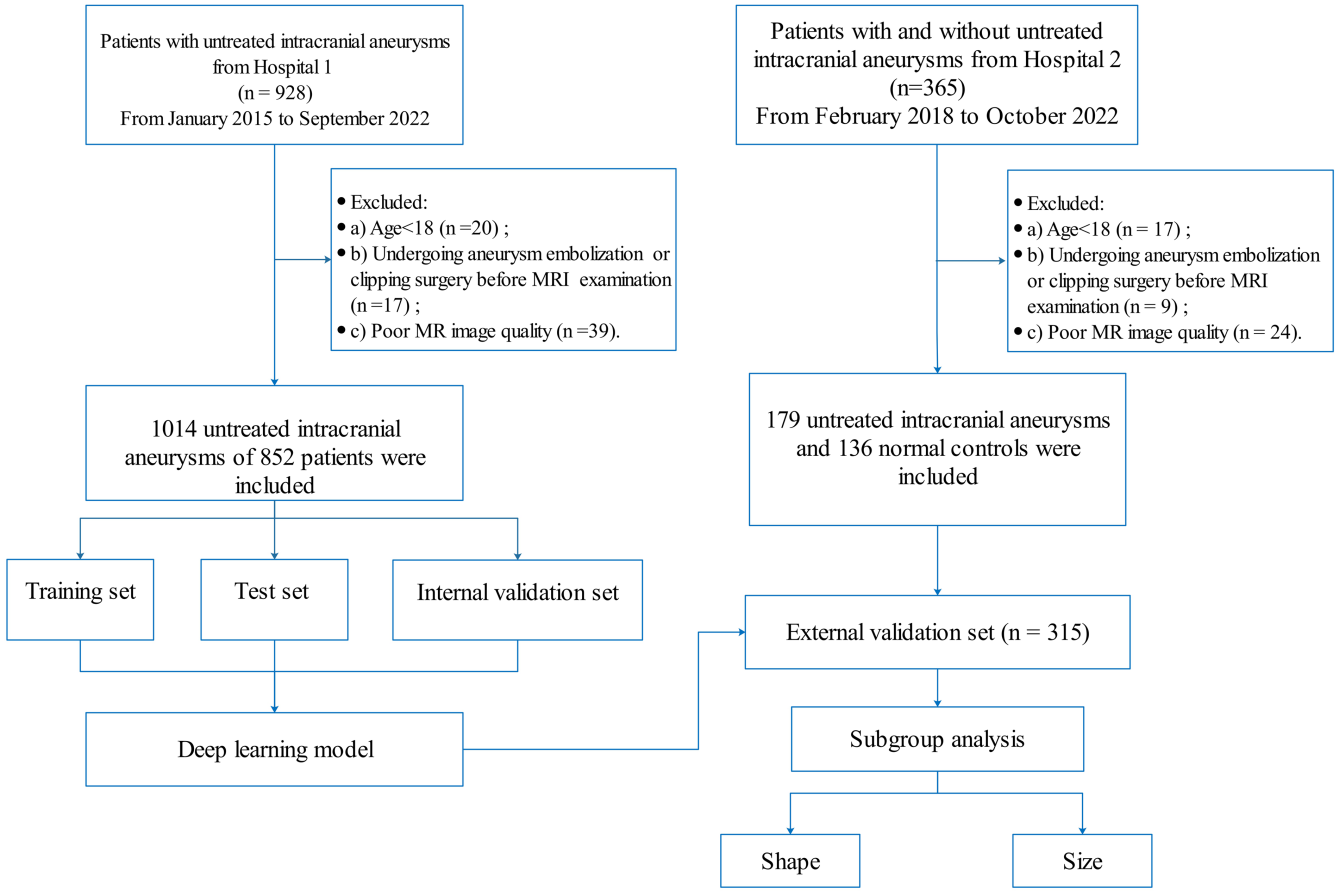
Flowchart of the patient enrollment.

A total of 928 patients with IA were retrospectively collected from hospital 1 between January 2015 and September 2022. All IAs were diagnosed by two radiologists with more than 8 years of brain MRI experience. If their diagnosis was inconsistent, a DSA examination was used for further confirmation. The inclusion criteria were as follows: (a) diagnosis of IA by MRA or (and) DSA and (b) the maximum diameter of IA was greater than 2 mm. The exclusion criteria were as follows: (a) age < 18 years, (b) poor MR image quality; and (c) undergoing aneurysm embolization or clipping surgery before MRI examination. All IAs were randomly divided into training, testing, and internal validation sets in a 7:2:1 ratio. In addition, 365 patients were enrolled from hospital 2 (including 179 patients with untreated intracranial aneurysms and 136 normal controls) between February 2018 and October 2022 for external validation. The flowchart of patient enrollment is shown in Figure 1.

### Clinical data collection MRA and DSA imaging

The clinical characteristics of age and sex were collected from the medical records. All MR images were obtained from four different MRI manufacturers. For the MRI systems, including the 3.0 T Magnetom Prisma, 3.0 T Trio Tim, and 1.5 T Magnetom Essenza by Siemens Corporation, the MRA data were obtained using the following parameters: repetition time (TR), 22 ms; echo time (TE), 3.69 ms; flip angle, 20°; field of view (FOV), 384 × 384; and section thickness: 0.6 mm. The scanning parameters of the T1WI sequence were as follows: TR, 153 ms; TE, 2.4 ms; FOV, 250 × 250; thickness, 5 mm; inter-slice gap, 1 mm; and flip angle, 70°. The scanning parameters of the T2WI sequence were as follows: TR, 5,560 ms; TE, 89 ms; FOV, 250 × 250; thickness, 5 mm; inter-slice gap, 1 mm; and flip angle, 150°. For an MRI of 3.0 T Achieva made by Philips Corporation, the MRA data were obtained with the following parameters: TR, 25 ms; TE, 3.45 ms; flip angle, 20°; FOV, 332 × 332; and section thickness: 0.6 mm. The scanning parameters of the T2WI sequence were as follows: TR, 532 ms; TE, 10.8 ms; FOV, 240 × 200; thickness, 5 mm; inter-slice gap, 2 mm; and flip angle, 150°. The scanning parameters of the T2WI sequence were as follows: TR, 4,569 ms; TE, 98.5 ms; FOV, 240 × 200; thickness, 5 mm; inter-slice gap, 2 mm; and flip angle, 180°. The DSA images were acquired from GE Innova IGS 5 and Innova 3100 with a rotational acquisition of 7.5 frames per second. Each DSA sequence consists of 15–30 frames with an image resolution of 1,024 × 1,024 pixels per frame.

### IA identification and deep learning

Two radiologists with over 8 years of experience manually and independently performed the IA identification and size measurement in the MRI and DSA scanning workstation. According to the morphology, all IAs were divided into regular and irregular subgroups. The maximum distance separating any two points on the surface of the IA was established as the maximum diameter, categorizing the IAs into three subgroups: < 3 mm, 3–5 mm, and > 5 mm.

A 3D CNN called MR 3D Unet was proposed for the automatic diagnosis and size measurements of IA from original 2D MR images. This MR 3D Unet was a symmetric encoder–decoder architecture that typically consists of four layers of encoders and four layers of decoders. A residual module in the encoder, instead of the original tiling structure, was used to extract the IA context information. Each layer of the encoder consists of two 3 × 3 × 3 convolutions + ReLU and a 2 × 2 × 2 max-pooling, which gradually downsamples and increases the number of channels to extract the high-level features, in which the number of convolutional channels in the four layers of the encoder is 64, 128, 256, and 512, respectively. The residual structure symmetric to the encoder was used in the decoder. Each layer of the decoder consists of a 2 × 2 × 2 deconvolution + jump-join (spliced to the features of corresponding layers of the encoder) and two 3 × 3 × 3 convolutions + ReLU, which gradually upsample and decrease the number of channels to recover the spatial details. The bottom layer connects the encoder to the decoder by two 3 × 3 × 3 convolutions, and the final segmentation result is output by a 1 × 1 × 1 convolution. Through the transformation of the decoder, the encoded features were extended to a full-resolution image with the same size and dimensions as the input volume. The advantage of using the residual model was that it ensured stable training even as the network depth increased significantly. Moreover, the fusion of different levels of features could be achieved through U-shaped skip connections. Most importantly, to increase the receptive field of the network, the dilated convolutions were used in the encoder’s top layer. The benefit of dilated convolutions lies in their ability to expand the receptive field without sacrificing information, thereby ensuring that each convolutional output encompasses a broader spectrum of data. Dilated convolutions could extract more global information and contextual features from the original 2D MR images.

**Figure 2:**
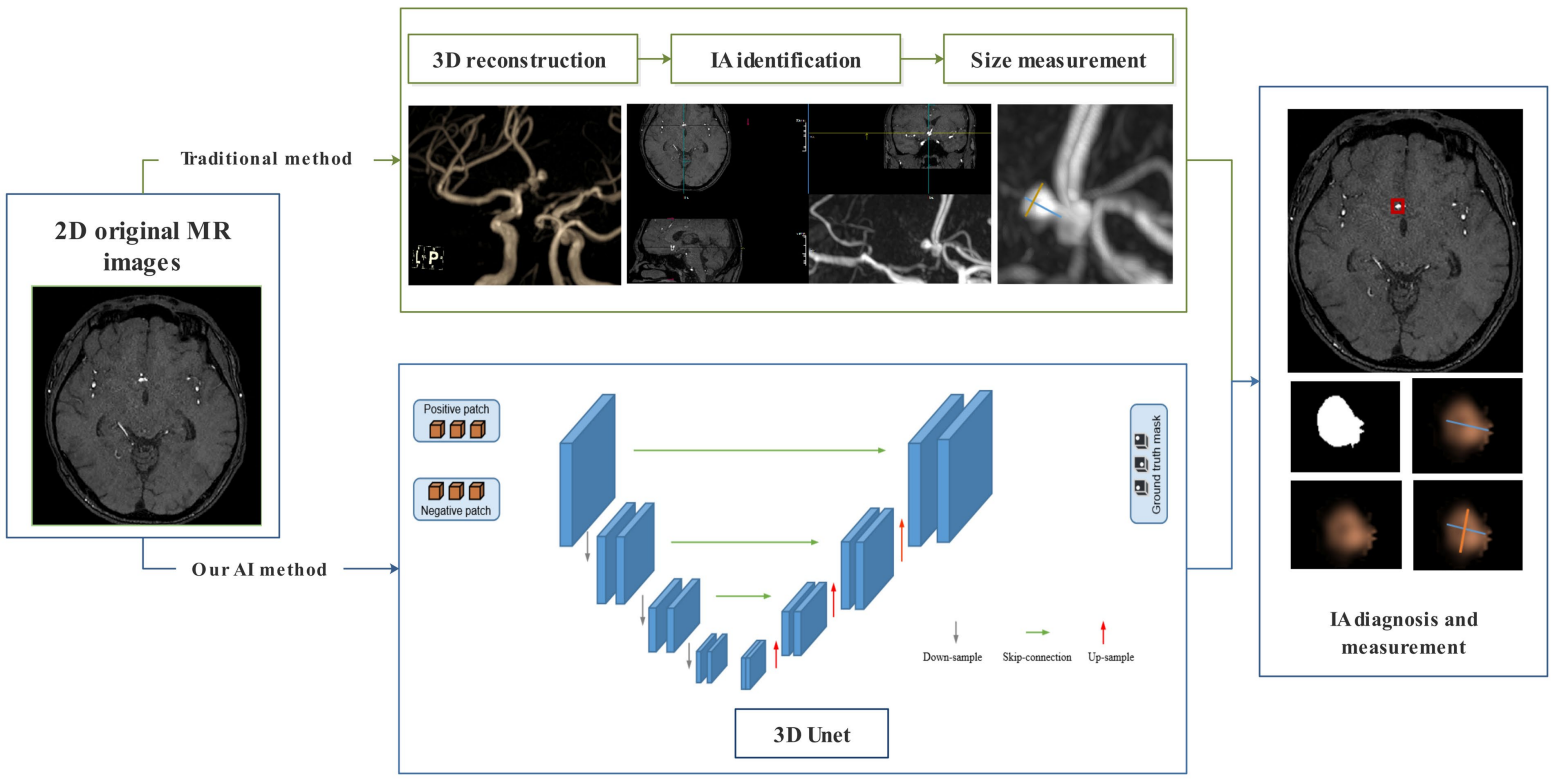
Flowchart of the traditional manual method and our automatic deep learning algorithm for IA diagnosis and measurement.

In our MR 3D Unet, the input volume of the original MR image was set to 80*80*80. To balance the data distribution, we sampled patches containing aneurysms as positive samples and patches without aneurysms as negative samples. Before these samples entered the network, we performed data augmentation, including rotation, flipping, and scaling. The pixel values of the patches were normalized to between 0 and 1. Our MR 3D Unet was implemented using PyTorch. Initially, a learning rate of 0.001 was used, which dropped to half every 25 epochs. The model was trained for 200 epochs using mini-batch gradient descent with a momentum of 0.9, and the average binary cross-entropy loss and Dice loss were used as the loss functions to optimize the network. In the test stage, the patches were generated by sequential sampling in the original MR image. Long and short diameters of the target IA were automatically calculated. The long diameter was defined as the maximum distance between any two points within the IA dome. The short diameter was defined as the maximum distance perpendicular to the long diameter (Figure 2).

### Statistical analysis

Multiple parameters, including true positive (TP), false positive (FP), false negative (FN), recall, sensitivity, and specificity indices, were used to assess the diagnosis performance of the MR 3DUnet. A two-sample *t*-test was employed to compare the size measurements obtained from MR3DUnet and radiologists. Inter-observer agreement between the two radiologists was quantified using the intraclass correlation coefficient (ICC). ICC > 0.75 means good consistency. A subgroup analysis of the aneurysm diagnosis and size measurement based on DSA was additionally performed. The data analysis was performed using R software (version 4.3.2^1^).

## Results

The demography of the included patients is shown in Table 1. A total of 852 patients (1,014 IAs) from hospital 1 were used for the training, testing, and internal validation sets. A total of 178 patients had multiple independent IAs: 168 patients (19.7%) had 2 lesions, and 10 patients (1.2%) had 3 lesions. The locations of IAs were as follows: anterior communication artery and anterior cerebral artery (ACCA) (98 cases, 9.7%), internal carotid artery (ICA) (777 cases, 76.6%), middle cerebral artery (MCA) (77 cases, 7.6%), and posterior circulation artery (PCA) (62 cases, 6.1%). A total of 315 patients (including 179 patients with untreated intracranial aneurysms and 136 normal controls) from hospital 2 were included as independent validation. Among 18 patients with multiple independent IAs, 17 patients (9.5%) had two lesions and 1 patient (0.6%) had 3 lesions. The distribution of IAs was as follows: ACCA (10 cases, 5.05%), ICA (174 cases, 87.9%), MCA (10 cases, 5.05%), and PCA (4 cases, 2.0%).

**Table 1:** Characteristics of patients in hospital 1 and hospital 2.

	Hospital 1	Hospital 2
Variables		
Age	58.04 ± 12.54	49.07 ± 10.51
Sex		
Male	248 (29.1%)	169 (53.7%)
Female	604 (70.9%)	146 (46.3%)
Morphology		
Regular	787 (77.6%)	165 (83.3%)
Irregular	227 (22.4%)	33 (16.7%)
Size		
<3 mm	366	38
3–5 mm	420	101
>5 mm	228	59
Location		
ACCA	98 (9.7%)	10 (5.05%)
ICA	777 (76.6%)	174 (87.9%)
MCA	77 (7.6%)	10 (5.05%)
PCA	62 (6.1%)	4 (2.0%)

**Figure 3:**
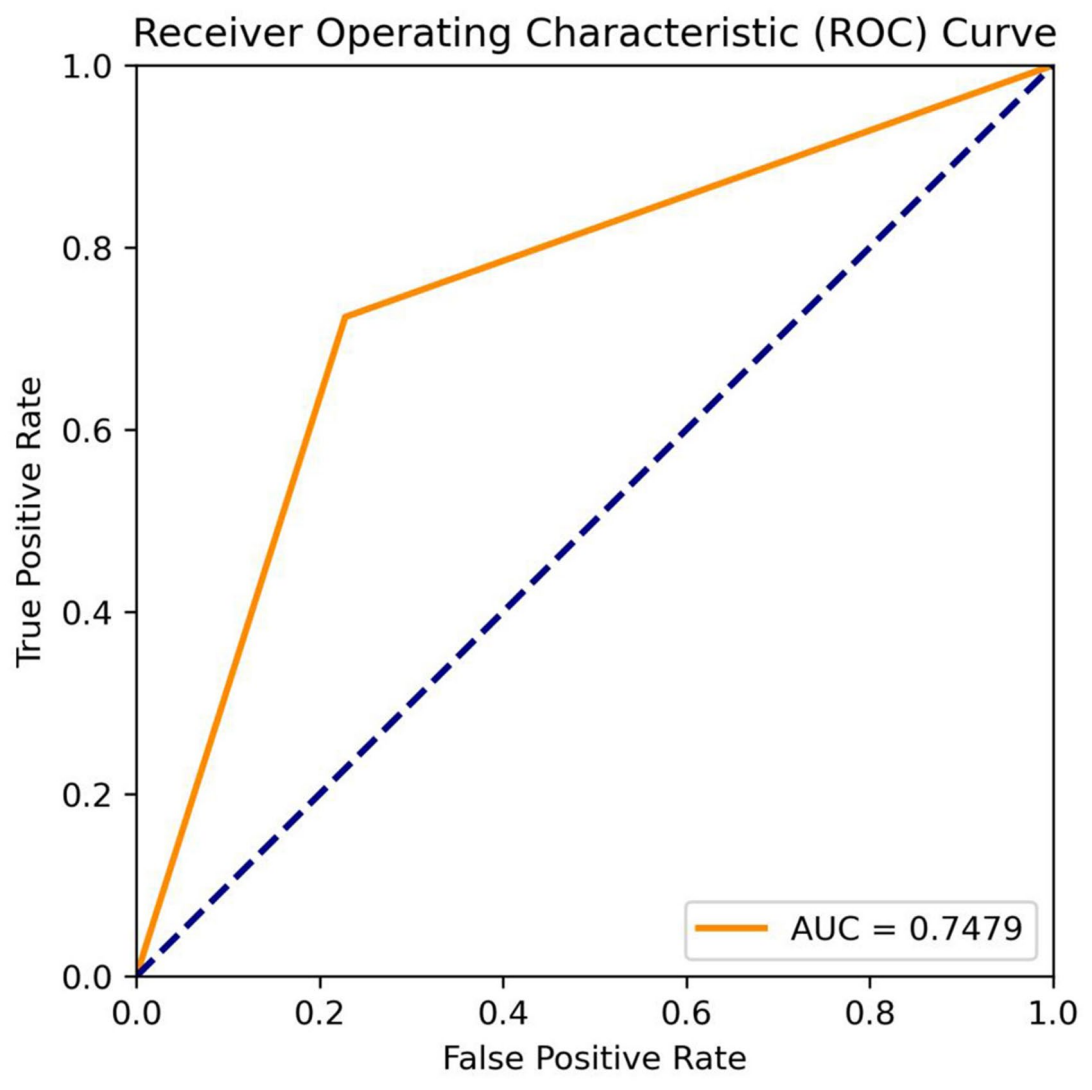
ROC curve and AUC value.

The fully automatic model could quickly process the original MRA data in 13.6 s and obtain real-time results, including IA diagnosis and size measurement. For the IA diagnosis, in the training, testing, and internal validation sets, the recall rates of the MR 3DUnet were 0.80, 0.75, and 0.79, and the sensitivities were 0.82, 0.75, and 0.75, respectively. In the independent validation set, the recall rate, sensitivity, specificity, and AUC were 0.71, 0.74, 0.77, and 0.75, respectively (Table 2; Figure 3). For the IA size measurement, no significant difference was found between our MR 3DUnet and the manual measurements of the two radiologists. The MR 3DUnet measured the long and short diameters of the IAs as 6.27 ± 2.43 mm and 4.62 ± 1.79 mm, respectively. The long and short diameters measured manually on MRA were 5.10 ± 2.66 mm and 3.81 ± 1.86 mm, respectively. The intraclass correlation coefficient (ICC) showed good consistency between the two radiologists (ICC > 0.75).

**Table 2:** Diagnostic performance of our deep learning model.

	Patients	Aneurysms	TP	FP	FN	Recall	F1 score	Precision	Sensitivity	Specificity
Training set	614	750	598	291	152	0.8	0.73	0.67	0.82	
Internal validation set	79	89	70	43	19	0.79	0.69	0.62	0.75	
Test set	159	175	132	122	43	0.75	0.62	0.52	0.75	
Independent validation	315	198	140	65	58	0.71	0.69	0.68	0.74	0.77

TP: true positive; FP: false positive; FN: false negative. Recall: lesion-level recall. Sensitivity: patient-level sensitivity. Specificity: patient-level sensitivity.

In the independent validation set, DSA data were available for 67 patients (74 aneurysms) out of 179 patients with IAs. Subgroup analysis showed a recall rate of 0.74 for IA diagnosis based on the DSA standard. The recall rate for irregular IAs was 0.73, while for regular IAs it was 0.75. The recall rates for IAs with sizes of <3 mm, 3–5 mm, and > 5 mm were 0.69, 0.79, and 0.70, respectively (Table 3). The long and short diameters measured manually on DSA were 6.27 ± 2.43 and 5.10 ± 2.66, respectively. The intraclass correlation coefficient (ICC) showed good consistency between the two radiologists (ICC > 0.75).

**Table 3:** Results of subgroup analysis of the aneurysms diagnosis based on DSA.

Subgroups		TP	FN	FP	Recall
Diagnosis based on DSA		55	19	31	0.74
Morphology	Regular	39	13		0.75
	Irregular	16	6		0.73
Size	<3 mm	9	4		0.69
	3–5 mm	27	7		0.79
	>5 mm	19	8		0.70

## Discussion

In the current clinical study, the 3D reconstruction, IA identification, and size measurement of MRA were very time-consuming and labor-intensive. A reliable artificial intelligence tool could greatly reduce the workload of radiologists and improve the efficiency of clinical diagnosis. In this research, we developed a deep learning algorithm utilizing the 3DUnet architecture for automatic diagnosis and size measurement of IA directly from the original 2D MR images. In terms of diagnosis, the recall rate, sensitivity, and specificity were 0.71, 0.74, and 0.77, respectively, in the external test set. In terms of size measurement, the results of our model showed no significant difference from the manual measurements taken by the two radiologists. To our knowledge, this is the first deep learning tool capable of automatically detecting and measuring IAs in raw 2D MRA data. The fully automatic “one-click” model could quickly process the original data within 15 s and obtain real-time results with high accuracy. It has the potential to significantly improve medical efficiency and reduce the workload of radiologists in clinical practice.

In this study, our 3DUnet was proposed based on the CNN model. The Unet was used because its architecture, with encoder and decoder blocks, offers high efficiency and accuracy, effectively extracting features from the global and local domains (21–23). The CNN algorithm based on this architecture provided pixel-level estimation, which could be used for detailed spatial prediction, such as the size of aneurysms. In addition, this network was very fast, and the segmentation of a 512 × 512 image took less than 1 s on the recent GPU (24). Our “one-click” 3DUnet model had many advantages compared to previously reported deep learning models. Previously, Nakao et al. (19) established a deep learning model that could detect IA using a “2.5D” net (applying the 9 directions of MIP images to cellular neural networks). Joo and Choi (20) developed a deep learning model combining 3D Unet and 3D ResNet, which could detect IA in approximately 1 min. Stember et al. (25) successfully developed a CNN for IA detection and size measurement from magnetic resonance MIP images. However, these previous models were all based on 3D images, requiring radiologists to first perform 3D reconstruction on the original image. Additionally, most of these models lacked automatic measurement functions, requiring doctors to manually measure the size of the aneurysm. This greatly reduced work efficiency and reduced its clinical value.

In the subgroup analysis, we found that our 3D Unet model showed a good ability for aneurysms of different shapes, indicating that the shape had minimal impact on the model’s accuracy. However, it is worth noting that the size of the IA significantly affected the performance of the model. The recall rate for aneurysms (<3 mm) was relatively lower, which aligns with previous studies (15, 20). For IA diagnosis, the recall of our algorithm was not very high. The possible reason was that most of the included IAs were small in size. In this study, small aneurysms (<3 mm) accounted for 33% of all IAs, which was much higher than those in previous studies (approximately 11%) (16, 26). On the other hand, to enhance the generalization of the model, our data were included from two different hospitals and four different MRI manufacturers. This might reduce the accuracy of the model but increase its reliability.

This study had several limitations. First, it was retrospective. Prospective studies with long-term longitudinal follow-ups could help further validate our model. Second, due to the continuous collection of all cases, the numbers of aneurysms in MCA, ACCA, and PCA were imbalanced. Finally, our algorithm was trained using unruptured aneurysms and might not be suitable for ruptured aneurysms. In the future, we will strive to overcome these limitations to improve and validate the accuracy of the model using larger samples and prospective studies.

## Conclusion

In this study, a one-click fully automatic deep learning model was developed for automatic IA diagnosis and size measurement based on 2D original MR images. It has the potential to significantly improve doctors’ work efficiency and reduce patients’ examination time, making it highly valuable in clinical practice.

## Data Availability

The data analyzed in this study is subject to the following licenses/restrictions: The materials and data are available from the corresponding author on specific request. Requests to access these datasets should be directed to Chuanming Li, licm@cqu.edu.cn.

## Footnotes

^1^ http://www.r-project.org
